# Amelioration of obesity-induced diabetes by a novel autophagy enhancer

**DOI:** 10.15698/cst2018.07.146

**Published:** 2018-06-13

**Authors:** Hyejin Lim, Myung-Shik Lee

**Affiliations:** 1 Severance Biomedical Science Institute and Dept. of Medicine, Yonsei University College of Medicine, Seoul, Korea.

**Keywords:** Tfeb, calcineurin, lysosome, Ca^2+^, phosphatase

## Abstract

Autophagy insufficiency due to aging, high-fat injury or genetic predisposition could be a factor in the progression of metabolic syndrome and diabetes. On the other hand, autophagy enhancement may have beneficial metabolic impact on *in vivo* metabolism of obese subjects. To identify novel, autophagy enhancer small molecules, we screened a chemical library using a Renilla-LC3-based luciferase assay [Lim *et al*. Nat Commun 9:1438]. Of the >7000 tested substances, one chemical compound, termed MSL (4-(4-fluorophenyl)sulfonyl-5-methylthio-2-phenyloxazole), (i) enhanced autophagic activity through Tfeb activation, (ii) expedited lipid clearance, probably through lipophagy, and (iii) reduced inflammasome activation through amelioration of mitochondrial dysfunction both *in vitro* and *in vivo*, leading to improved metabolic profile of mice with genetic or diet-induced obesity.

Our screening system was a convenient high throughput assay of autophagic flux rather than cumulative autophagy levels, and the index of autophagic activity was calculated as the ratio of the luciferase fluorescence of an autophagy-susceptible construct to that of an autophagy-resistance one. Among the 35 chemicals enhancing autophagic activity, we selected 7 that do not inhibit mTORC1 since prolonged mTORC1 inhibition may have adverse metabolic effects such as decrease of pancreatic β-cell mass. Among them, we found that three chemicals improved the metabolic profile of obese mice *in vivo* in our preliminary test. We focused on one of the 3 chemical showing *in vivo* effects (MSL). We confirmed that autophagy activated by MSL progressed to the lysosomal step in a cell model using a tandem *mRFP*-*GFP*-*LC3 *construct, and found that MSL increases lysosomal content as demonstrated by acridine orange staining. In our effort to elucidate the cause of the increase in lysosomal content, we observed that MSL induces prominent nuclear translocation of Tfeb, a master regulator of lysosomal biogenesis and autophagy gene expression. We also found that MSL reduces phosphorylation of S142 of Tfeb, one of the most important phosphorylation sites of Tfeb. Since MSL did not inhibit the Tfeb regulator mTORC1, we searched for an alternative mechanism of reduced S142 phosphorylation, and found that MSL increases activity of calcineurin, which is the most important phosphatase in the starvation-induced activation of Tfeb. However, in contrast to starvation-induced Tfeb activation, concomitant perilysosomal Ca^2+^ release was not observed. Instead, MSL appeared to induce direct activation of calcineurin. Binding of MSL to calcineurin was substantiated by a drug affinity responsive target stability (DARTS) assay which showed altered susceptibility of calcineurin to pronase, presumably due to MSL binding to calcineurin. A crucial role of calcineurin activation in autophagy induction and Tfeb nuclear translocation was supported by the abrogation of MSL-induced Tfeb nuclear translocation by a dominant-negative mutant of calcineurin A subunit.

We next studied whether MSL could change cellular metabolism through autophagy activation. When cells loaded with a combination of palmitic acid (PA) plus oleic acid (OA) were treated with MSL, accelerated clearance of BODIPY^+^ lipid droplets was observed, which was abrogated by orlistat, a nonspecific inhibitor of lipases, or lalistat2, a specific inhibitor of lysosomal lipase. Furthermore, BODIPY fluorescence colocalized with Lamp1, a marker of lysosomes, and RFP-LC3, a maker of autophagolysosomes, suggesting occurrence of lipophagy mediated by lysosomal lipase. We also studied the effect of MSL on mitochondrial dysfunction of macrophages, which plays a crucial role in inflammasome activation and metabolic inflammation in metabolic tissues of obese mice or patients with diabetes. MSL was found to ameliorate mitochondrial dysfunction such as mitochondrial ROS production or mitochondrial potential loss after combined treatment with lipopolysaccharide (LPS) plus PA. Accordingly, inflammasome activation and release of mature IL-1β by LPS plus PA, were downregulated in MSL-treated macrophages, probably due to attenuated mitochondrial dysfunction.

We next studied *in vivo* effects of MSL on *ob/ob* mice, a classical genetic model of obesity. MSL administration markedly improved metabolic profile of *ob/ob* mice, leading to reduced nonfasting blood glucose level, enhanced glucose tolerance and reduced insulin resistance without changes of body weight. The fatty liver phenotype of *ob/ob* mice was also ameliorated as evidenced by the reduction of fat accumulation in the liver and of serum liver enzymes, which could be due to enhanced lipophagy in the liver. In adipose tissue, the number of crown-like structure (macrophage aggregates around dead adipocytes) representing metabolic inflammation, was reduced, which was accompanied by attenuated expression of cytokines and inflammasome activation in the stromal vascular fraction (**Figure 1**).

**Figure 1:**
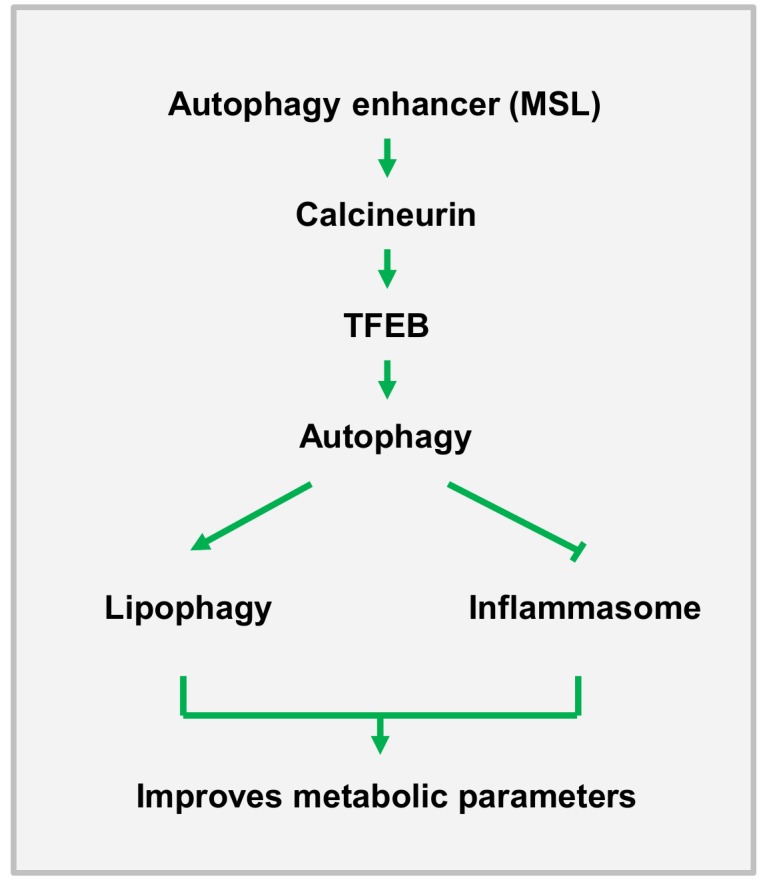
FIGURE 1: A diagram of the effect of autophagy enhancer on *in vivo* metabolism. MSL enhances autophagic activity through calcineurin activation, which leads to improved metabolic profile of obese mice by increasing lipophagy and suppressing inflammasome activation or metabolic inflammation.

While these results were encouraging, the effect of MSL on mice fed a high-fat diet (HFD) was only marginal, to our disappointment. We found that microsomal stability of MSL was suboptimal (< 10% remaining after 30 min incubation with liver microsome), which could be the cause of insufficient metabolic improvement by MSL in mice with diet-induced obesity. Thus, we made several chemical derivatives of MSL, and found one chemical (MSL-7) that has much improved microsomal stability (> 90% remaining after 30 min incubation with liver microsome), while retaining similar autophagy enhancing activity. Indeed, MSL-7 could significantly improve metabolic profile not only of *ob/ob* mice but also of mice fed HFD, suggesting the possibility that autophagy enhancer small molecules could be a new therapeutic agent against diabetes or metabolic syndrome with lipid overload or obesity. To confirm that the improvement of metabolic profile is due to autophagy induction or Tfeb activation, we administered dimethyl-α-ketoglutarate (DMKG), an autophagy inhibitor, and also conducted *in vivo* knockdown of Tfeb. Both DMKG and *in vivo* knockdown by injection of *siTfeb *together with Invivofectamine reversed metabolic improvement by MSL-7 administration (**Figure 2**), supporting that metabolic improvement by MSL-7 is due to activation of Tfeb and autophagy. We also studied whether autophagic activity is indeed induced by MSL administration *in vivo*. Conversion of LC3-I to -II in the liver after lysosomal clamping by leupeptin administration was increased after *in vivo* administration of MSL-7, showing increased auphagic flux. When the expression of potential target genes of Tfeb was examined, several autophagy genes (*Lc3, Beclin 1, Uvrag*) and lysosome genes (*Lamp1, Mcoln1, Clcn7, CtsA, and CtsD*) were induced in the liver, consistent with the activation of Tfeb by MSL-7 administration *in vivo*. The expression of *Tfeb* itself was increased after MSL or MSL-7 administration. In muscle, the expression of mitochondrial genes such as *Mfn1*, *Mfn2*, *Nrf-1*, *Nrf-2, Tfam*, *Cox1*, *Cox2* or *CS*) was additionally induced after *in vivo* MSL-7 administration. In adipose tissue, the expression of autophagy genes was elevated in mice fed HFD, which was not further enhanced by the autophagy enhancer. These results indicate that autophagy levels and its response to autophagy modulators are distinct, depending on the type of tissues, and that systemic metabolic response to autophagy modulators could result from integration and interaction of metabolic responses of metabolic organs responding differently to autophagy demand and stimuli.

**Figure 2:**
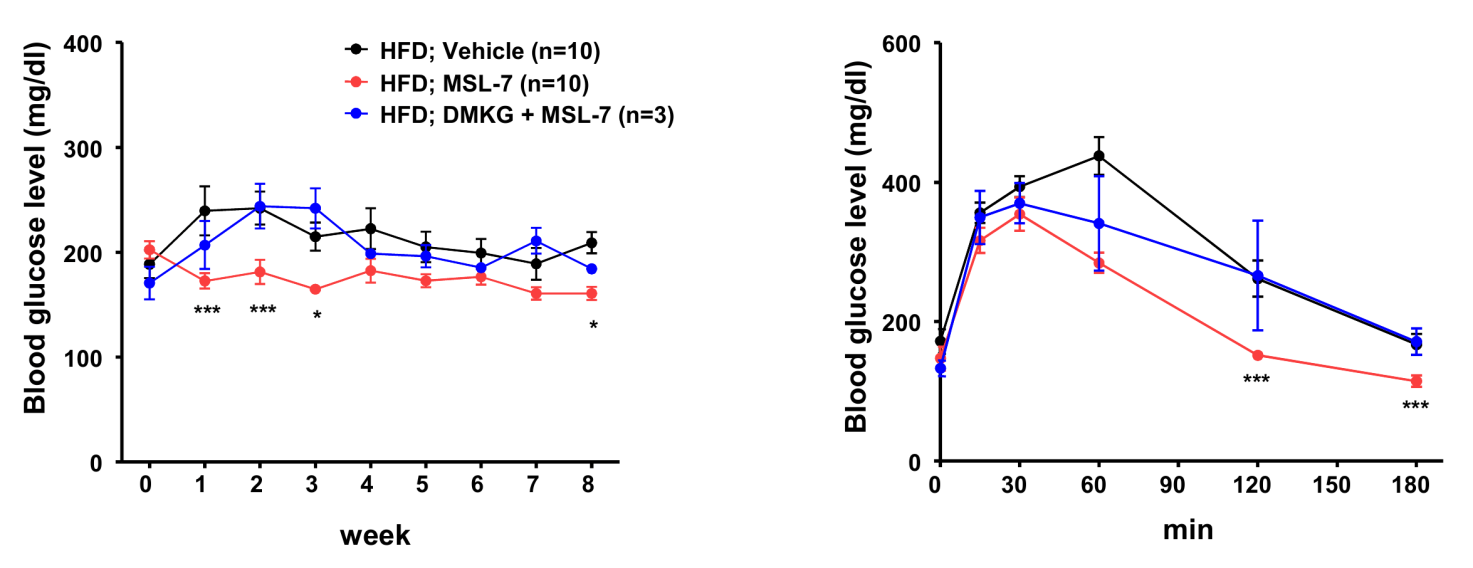
FIGURE 2: Reversal of MSL-7-induced metabolic improvement in HDF-fed mice by an autophagy inhibitor. MSL-7 was administered to mice fed HFD for 8 weeks with or without DMKG, an inhibitor of autophagy. Nonfasting blood glucose level was monitored during administration of MSL-7 and DMKG to HFD-fed mice for 8 weeks (left). Intraperitoneal glucose tolerance test was conducted after 8 weeks of administration (right).

Several questions could be raised from this study. First of all, potential adverse effects of chronic autophagy enhancement or Tfeb activation need to be carefully studied, although we could not detect abnormalities in major organ biopsies, blood chemistry or hemogram after 8 weeks of administration. Since Tfeb family members can act as oncogenes in some tissues particularly when their expression levels are extremely increased, enhancement of autophagic activity or Tfeb activity may need to be ‘appropriate’ to a certain level. Activity of non-selective or selective autophagy appears to be decreased in most, if not all, tissues in aging or after high fat intake, elevation of autophagy to ‘normal’ level observed in youth or after ‘normal’ diet could be a reasonable goal. It is also not clear whether calcineurin is a sole target of MSL or MSL-7. More studies to confirm direct physical binding of MSL to calcineurin and search for other potential target molecules would be necessary. The effect of MSL-mediated calcineurin activation on phosphorylation of Tfeb at sites other than S142 and that on phosphorylation of other Tfeb family members such as Tfe3 were also not clarified. In addition, the effects of calcineurin activation besides Tfeb or autophagy activation might be intriguing topics to be addressed. Calcineurin plays important roles in many physiological or pharmacological processes in diverse tissues. One obvious question is what the effects of prolonged calcineurin activation on innate or adaptive immunity are, as calcineurin inhibitors have long been used as immunosuppressants in organ transplantation. Indeed, we also observed that MSL reduces not only pro-IL-1β maturation to IL-1β but also the expression of pro-IL-1β, which may not be directly related to its effect on inflammasome activation. Moreover, release of TNF-α and IL-6 in response to LPS was reduced by MSL-7. Further studies disclosed that LPS-induced NF-κB activation was attenuated by MSL-7, which might be related to previously reported calcineurin binding to and inhibition of MyD88 and TRIF.

Finally, several practical issues need to be addressed before their application to clinical trials such as target affinity, bioavailability, solubility and pharmacokinetic behavior of autophagy enhancers. When these issues could be answered, development of autophagy modulators for clinical application to patients could become a reality.

